# Are body size and volatile blends honest signals in orchid bees?

**DOI:** 10.1002/ece3.2903

**Published:** 2017-03-23

**Authors:** Brenda Jessica Arriaga‐Osnaya, Jorge Contreras‐Garduño, Francisco Javier Espinosa‐García, Yolanda Magdalena García‐Rodríguez, Miguel Moreno‐García, Humberto Lanz‐Mendoza, Héctor Godínez‐Álvarez, Raúl Cueva del Castillo

**Affiliations:** ^1^ UBIPROLaboratorio de EcologíaFES IztacalaUniversidad Nacional Autónoma de MéxicoMéxico CityMéxico; ^2^ ENES, Unidad MoreliaUniversidad Nacional Autónoma de MéxicoMoreliaMichoacánMéxico; ^3^ Instituto de Investigaciones en Ecosistemas y SustentabilidadUniversidad Nacional Autónoma de MéxicoMoreliaMichoacánMéxico; ^4^ Centro de Investigaciones Sobre Enfermedades InfecciosasInstituto Nacional de Salud PúblicaCuernavacaMorelosMéxico

**Keywords:** honest signals, orchid bees, phenoloxidase, sexual selection

## Abstract

Secondary sexual traits may convey reliable information about males’ ability to resist pathogens and that females may prefer those traits because their genes for resistance would be passed on to their offspring. In many insect species, large males have high mating success and can canalize more resources to the immune function than smaller males. In other species, males use pheromones to identify and attract conspecific mates, and thus, they might function as an honest indicator of a male's condition. The males of orchid bees do not produce pheromones. They collect and store flower volatiles, which are mixed with the volatile blends from other sources, like fungi, sap and resins. These blends are displayed as perfumes during the courtship. In this study, we explored the relationship between inter‐individual variation in body size and blend composition with the males’ phenoloxidase (PO) content in *Euglossa imperialis*. PO content is a common measure of insect immune response because melanine, its derived molecule, encapsulates parasites and pathogens. Body size and blend composition were related to bees’ phenolic PO content. The inter‐individual variation in body size and tibial contents could indicate differences among males in their skills to gain access to some compounds. The females may evaluate their potential mates through these compounds because some of them are reliable indicators of the males’ capacity to resist infections and parasites.

## 1 Introduction

Male secondary sexual traits may convey reliable information about males’ health and condition (Zahavi, 1975). Hence, the evolution of exaggerated ornaments and sexual signals could be partiality explained because these traits can signal parasites or infection resistance, and females should prefer ornate mates because their genes for resistance would be passed on to their offspring (Andersson, 1994; Jacobs & Zuck, 2012). Resistance could be screened as a function of immune system, and given that there are evolutionary costs associated with the expression of sexually selected traits (SST; Jacobs & Zuck, 2012), and immune system (Demas, Greives, Chester, & French, 2012), those males showing the more elaborated SST should also be healthy (Jacobs & Zuck, 2012). This is explained because costs impose a handicap, and only males in good condition can canalize resources to both traits (Getty, 2002; Sheldon & Verhulst, 1996; Westneat & Birkhead, 1998). Thus, if resources must be diverted away from the immune system in order to maximize expression of a sexually selected trait, a trade‐off between sexual trait expression and immune function can evolve (Sheldon & Verhulst, 1996).

Several studies on insects have suggested that sexual characters do indicate male immunocompetence to females (Ryder & Siva‐Jothy, 2000; Siva‐Jothy & Thompson, 2002; Reviewed by Contreras‐Garduño & Canales‐Lazcano, 2013). Phenoloxidase (PO) content is one of the most common measured component of immune response related to sexual selection because its derived molecule, melanin, is a compound used to surround and encapsulate parasites and pathogens (reviewed by Contreras‐Garduño & Lazcano, 2013). During melanogenesis, PO catalyzes the hydroxylation of tyrosine to L‐dihydroxy phenylalanine (L‐DOPA) to produce the melanin precursor, indolequinone, that combats parasites and pathogens (Nappi & Vass, 1993). Quinoid compounds are potent catalysts of the production of reactive oxygen molecules (ROS; Nappi, Vass, & Carton, 1995) and the oxidation of L‐DOPA can generate superoxide anion and hydrogen peroxide, which are toxic molecules to parasites (Nappi & Vass, 1993; Nappi et al., 1995).

In many insect species, larger males have high mating success (Alcock & Thornhill, 1983) and can canalize more resources to the immune function (Córdoba‐Aguilar, Jiménez‐Cortés, & Lanz‐Mendoza, 2009; Ryder & Siva‐Jothy, 2000). Moreover, several species use pheromones produced in specialized secretory glands to identify and attract conspecific mates. Species‐specific pheromone signals are important in the recognition functions for pre‐mating isolation (Wyatt, 2003; Wyatt, 2010), while other pheromones can function as an honest indicator of a male's resistance to parasites and, in general, male condition (Penn & Potts, 1999; Rantala, Jokinen, Kortet, Vainikka, & Suhonen, 2002; Rantala & Kortet, 2003). Some pheromone components could be the signals of resistance, and therefore, immune response, providing information about the evolution and significance of secondary sexual traits (Candolin, 2008; Iwasa & Pomiankowski, 1994). However, as far as we know, there are no specific relationships between different male pheromones compounds as sexual traits with immune response (reviewed in Contreras‐Garduño & Lazcano, 2013). Nonetheless, the relationship between pheromones and immune resistance has been tested in one insect species, *Tenebrio molitor* (Rantala et al., 2002).

The males of orchid bees (Apidae, Euglossini), a group of long–tongued tropical bees, collect and store flower volatile blends, which are mixed with volatiles from other sources and displayed as perfumes during the courtship (Eltz, Sager, & Lunau, 2005). Orchid bees store these volatiles in specialized, enlarged hind–tibial pouches (Williams & Mark, 1983). The blends are composed mostly of terpenoids and aromatic volatiles (Dressler, 1982; Eltz, Whitten, Roubik, & Linsenmair, 1999; Williams & Mark, 1983). The orchid bees collect these compounds by a process known as fatty extraction. A small quantity of lipoid cephalic gland secretions is placed on the fragrant surface and used to transfer the volatile compounds to the hind leg pouches (Eltz et al., 2007; Whitten, Young, & Williams, 1989). Flowers of orchid species produce scents that specifically attract males of one or few bee species. As males collect scents from these flowers, they act as their exclusive pollinators (Dressler, 1982). Because many of the collected compounds are toxic to bees and difficult to find, it has been suggested that males are carrying a handicap that could show to the females their endurance to toxicity and their capacity to survive and collect scarce resources (Roubik & Hanson, 2004).

In this study, we explored the relationship between inter‐individual variation in body size and blend composition with the males’ phenoloxidase (PO) content in *Euglossa imperialis* collected in Los Tuxtlas, Veracruz, Mexico. *Euglossa imperialis* is distributed in tropical regions of South America, Central America (Nemésio & Silveira, 2007), and Mexico. In Los Tuxtlas, the species can be found all year round. We tested the hypothesis that male size and blends are honest signals of their immune response. We expected a positive relationship between body size and PO content, and a significant relationship between blend composition and PO content.

## 2 Methods

### 2.1 Sampling procedure

Males from *Euglossa imperialis* and other orchid bee species were collected in the neighboring areas of the Tropical Biology Field Station of Los Tuxtlas, Veracruz, Mexico. The location is a rain forest area located next to the Gulf of Mexico (18°34′–18°36′N, 95°04′–95°09′W, 150 m above sea level), with an annual precipitation of 4,500 mm. Two 6‐day sampling periods were carried out in the first week of April and the last week of June, 2013. Three collectors captured males of *Euglossa imperialis* and other orchid bees with entomological nets (BioQuip 7325NA) baited with cineole, L– carvone, and methyl salcilate in 300‐m paths around the field station from 09:00 to 14:00 h. Cineole and methyl salcilate are general euglossine attractants (Roubik & Hanson, 2004). Captured bees were placed in individual plastic bags (7 × 5 cm) to measure the thorax and head width with a digital caliper (Mitutoyo Corp., Tokyo, Japan). Later, they were placed in their plastic bags and stored in liquid nitrogen in a 5‐L nitrogen thermo (MVEsc; USA). In the laboratory, the hind legs of each individual were dissected and extracted in 0.5 ml of hexane for analysis. Before conducting further analyses, the thorax and head width data were ordinated using a principal components analysis.

### 2.2 Immunocompetence capacity

We evaluated the immunocompetence capacity through the PO content in the bees’ body. Phenoloxidase (PO) content has previously been recorded in several species of insects, including other bee species (Adamo, 2004; Gershman et al., 2010; Srygley, Lorch, Simpson, & Sword, 2009). To estimate PO content, the bee's thorax was macerated in 1 ml of phosphate buffered saline (PBS; Sigma). Then, we took one sample of 40 μl and deposited it in a microplate template of 96 round‐bottomed wells (Corning) containing 60 μl of PBS and 100 μl of L‐DOPA diluted in PBS (4 mg/ml). PO content was determined indirectly by oxidation of 3,4‐dihydroxy‐L‐phenylalanine (L‐DOPA, Sigma). One sample of 100 μl of PBS and 100 μl of L‐DOPA (4 mg/ml) were used as blanks. The slope of the curve was calculated using the optical density at 490 nm with an ELISA reader (Microplate Reader Series, BMGLABTECH). Optical density readings were taken every 5 min during one hour at 27°C.

### 2.3 Chemical analysis

A random subsample of 100 *Euglossa imperialis* males was used to analyze their blend composition. One microliter of each hexane extract was analyzed using an Equity 5 capillary column (30 m × 0.25 mm ID × 0.25 μm film thickness; 5% phenyl methylsiloxane) in an Agilent 6890 gas chromatograph (GC) coupled to an Agilent 5973N mass selective detector. The injection was split (60.3:1) at 220°C. Helium was used as carrier gas at a constant flow of 1.0 ml/min. Electronic flow control (EFC) was used to maintain a constant helium carrier gas flow of 1 ml per min. The GC oven temperature was held at 60°C, and then increased by 5°C per min to 300°C. The mass spectrometer (MS) interface was at 280°C and the ion trap worked at 230°C. The MS was set at 70 eV (in EI mode) with a scanning speed of 2.94 scans per second, from 40 to 500 m/z. Data were collected and integrated using the Environmental Chemstation software from Agilent Technologies. The identification of a compound was considered valid when its chromatographic peak was pure, its mass spectrum coincided with that of a known compound in the National Institute of Standards and Technology (NIST02) library, and the calculated Kovats index was close to the published Kovats index for the same kind of column used in our analysis. The chemical identification of some components was confirmed by injection of known standards purchased from Sigma Aldrich (Table 2). The relative concentration of each compound was estimated by dividing its peak area by the sum of the areas of all peaks detected in the sample (total peak area). Moreover, we estimated the amount of the blend for each bee adding up the areas of all compounds in the bee's hind leg. The inter‐individual variation of the blend composition was summarized using a principal components analysis. The first three principal components explained 48.8% of the variation in blend composition (Table 2) and were used in further analyses. Because the availability of blend sources can change temporarily, the blend composition from bees collected in April and June was compared.

### 2.4 Relationship between phenoloxydase content and male body size and blends composition

To explore the relationship between PO content and male's body size, we regressed PO content on males body size (PC 1; see Section 2) considering all males captured in the field. The PO content values were log‐transformed previous to the analysis in order to improve the normality assumptions of parametric tests. To remove body size effects on PO content, the residuals of the regression were used to conduct simple linear regressions between the PO content and number of compounds, amount and the blends complexity. These regressions were conducted separately for April and June, because we found significant differences in the complexity and amount of blends between these months and in the PC 2 of blend composition (see Section 3). In addition, multiple linear regression was performed to analyze the relationship between the PCs 1 and 2 of blend composition, and PO content residuals. Simple and multiple regressions were conducted with a random subsample of one hundred individuals in which the blend composition was analyzed (see Section 2.3).

## 3 Results

### 3.1 Males’ body size

A total of 275 males of *Euglossa imperialis* were captured. They had an average thorax width of 5.31 mm (*SE* = 0.017) and an average head width of 5.16 mm (*SE* = 0.017). The first principal component (PC 1) explained 85% of variance of thorax and head width, and was considered as a body size index because the eigen vectors of the two morphological characters were positive (Table 1).

**Table 1:** Principal component analysis of thorax and head width of 275 males of *Euglossa imperialis* collected at Los Tuxtlas, Veracruz, Mexico

Trait	PC 1	PC 2
Thorax width	0.707	−0.707
Head width	0.707	0.707
Eigenvalue	1.674	0.352
% of total variance	83.74	16.26

### 3.2 Blend composition

We found 60 compounds in one hundred males of *Euglossa imperialis* (Table 2). Fifty‐six of these bees were collected in April and 44 in June. The average male had 24.4 of these compounds in its blend. The most represented compounds were tetradecyl tetradecanoate (87%), β‐cubebene (76%), Z,E‐2,13‐octadecadien‐1‐ol (65%), and eicosane (61%), respectively. Besides, the compounds allo‐aromandrene, phenylethyl alcohol, and τ‐terpinene were found each one in just one individual. Terpinolene was just found in males captured on April, whereas three compounds: Benzyl alcohol, β‐linalool, and menthol were just found in bees captured in June.

**Table 2:** Chemical compounds identified in hind legs of *Euglossa imperilis* males collected in “Los Tuxtlas,” Veracruz, Mexico

Compound	RI^a^	RI^b^	No. males
α‐Pinene^a^	921	932	29
Camphene^a^	937	946	18
Sabinene	959	969	23
β‐Pinene^a^	965	974	21
β‐Myrcene	972	988	27
α‐Phellandrene^a^	989	1,002	14
3‐Carene^a^	998	1,008	12
p‐Cymene	1,009	1,020	8
Limonene^a^	1,011	1,024	20
Eucalyptol	1,014	1,026	29
β‐Ocimene	1,024	1,032	21
Benzyl alcohol	1,039	1,033	7
γ‐Terpinene^a^	1,047	1,054	1
Terpinolene	1,056	1,086	3
Phenylethyl alcohol	1,059	1,083	1
Linalool	1,065	1,095	12
Menthol	1,145	1,167	5
4‐Terpineol^a^	1,155	1,184	6
α–Terpineol	1,173	1,186	7
Estragole	1,184	1,195	22
Methyl salicylate^a^	1,192	1,190	24
Ethyl salicylate	1,206	1,267	35
Thymol^a^	1,282	1,289	5
Undecanal	1,312	**1,310**	11
E,E‐ 2,4‐Decadienal	1,324	**1,330**	9
Eugenol	1,343	1,356	15
Geranylacetate	1,382	1,379	12
α‐Gurjunene	1,400	1,409	11
β‐Caryophyllene^a^	1,414	1,417	24
β‐Cedrene	1,420	1,419	16
β‐Gurjunene	1,438	1,431	33
α‐Humulene^a^	1,448	1,452	10
Allo‐Aromandrene	1,457	1,458	1
Germacrene D	1,475	1,484	76
Ledene	1,478	1,496	18
α‐Farnesene	1,482	1,505	24
β‐Bisabolene	1,488	1,506	22
δ ‐Cadinene	1,531	1,522	23
Epiglobulol	1,538	1,564	21
(‐)‐Globulol	1,546	1,584	19
Ledol	1,574	1,602	13
α‐Bisabolol	1,718	1,685	41
E,E‐Farnesal	1,744	1,740	22
Hexahydrofarnesylacetone	1,848	1,847	37
Hexadecanoic acid, methyl ester	1,934	1,933	53
Hexadecyl acetate	2,016	2,010	43
Methyl oleate	2,098	2,085	26
Octadecanoic acid, methyl ester	2,109	2,112	13
Linoleic acid	2,111	2,113	18
Methyl octaclecanoate	2,121	2,124	43
Oleic acid	2,142	2,141	49
Ethyl Oleate	2,156	2,174	29
(E)‐9‐Octadecenoic acid ethyl ester	2,189	2,185	29
Z‐13‐Octadecen‐1‐ol acetate	2,204	2,200	45
(Z)‐9‐Tricosene	2,259	2,271	33
Heptacosane	2,713	2,700	56
Squalene	2,763	2,757	52
Unknown 1	2,989	NA	87
Triacontane	3,008	3,000	61
Unknown 2	3,059	NA	65

NA: RI not available.

The designed number in the males column indicates the number of insects that contained the metabolite in a group of 100 males. Kovats Retention Index (RI^a^) on a HP‐5MS capillary column obtained experimentally, Kovats RI^b^ retention index available in the literature.

a Compounds that were identified by the comparison with the retention times and mass spectra of the authentic commercial compounds (Adams, 2007; Linstrom & Mallard, 2001).

The first principal component (PC 1) explained 24.83% of variance of the blend composition, the second explained (PC 2) 13.77% and the third (PC 3) explained 10.19%. Together the three PCs explained 48.8% of the variance in the blend composition (Table 3) and were used in further analysis. The compounds with highest relative weight on the PC 1 were α‐bisabolol (0.215), E,E‐farnesal (0.198) and (E)‐9‐ Octadecenoic acid ethyl ester (0.192). The eigenvalues for the compounds allo‐aromandrene, terpinolene and τ‐terpinene were slightly negative, indicating that their proportions decrease when other compounds were abundant. In the PC 2, the eigenvalues of 33 compounds showed a positive relationship, and 27 were negative. Tetradecyl tetradecanoate (0.239) and oleic acid (0.239) showed the highest positive weight on the PC 2, Z‐13‐octadecen‐1‐ol acetate (−0.209), and estragole (−0.185) had the highest negative weight. For the PC 3, the eigenvalues of 25 compounds showed a positive relationship, and 35 were negative. Eicosane (0.332), (‐)‐globulol (0.330) and camphene (0.321) showed the highest positive weight, and α pinene (−190), β‐pinene (−0.187), and D‐limonene (−0.182) had the highest negative weight.

**Table 3:** Principal component analysis of 60 compounds found in 100 males of *Euglossa imperialis* collected at Los Tuxtlas, Veracruz, Mexico. Only the eigenvalues of the first three Principal Components (PC) and their explained variance are shown

Compound	PC 1	PC 2	PC 3
α‐Pinene	0.124	0.135	−0.190
Camphene	0.111	0.034	0.321
Sabinene	0.118	0.180	−0.063
β‐Pinene	0.137	0.098	−0.187
β‐Myrcene	0.118	0.191	−0.167
α‐Phellandrene	0.125	0.190	−0.158
3‐Carene	0.164	0.052	−0.039
p‐Cymene	0.136	0.100	−0.088
Limonene	0.117	0.147	−0.182
Eucalyptol	0.074	0.103	−0.119
β‐Ocimene	0.105	0.227	−0.081
Benzyl alcohol	0.145	0.025	−0.051
γ‐Terpinene	−0.020	−0.002	0.003
Terpinolene	−0.018	0.013	−0.001
Phenylethyl alcohol	0.030	0.067	0.026
Linalool	0.171	−0.177	−0.048
Menthol	0.172	−0.133	−0.073
4‐Terpineol	0.090	0.036	0.112
α–Terpineol	0.125	−0.014	0.171
Estragole	0.148	−0.185	−0.053
Menthyl salicylate	0.174	−0.117	−0.098
Ethyl salicylate	0.113	0.074	−0.057
Thymol	0.101	−0.063	0.032
Undecanal	0.177	0.038	0.030
E,E‐ 2,4‐Decadienal	0.186	−0.177	0.003
Eugenol	0.159	−0.160	−0.024
Geranylacetate	0.001	−0.050	−0.010
α‐Gurjunene	0.006	−0.014	0.010
β‐Caryophyllene	0.052	0.127	0.005
β‐Cedrene	0.138	0.014	0.233
β‐Gurjunene	0.108	−0.020	0.142
α‐Humulene	0.153	0.057	0.252
Allo‐Aromandrene	−0.023	−0.002	0.004
Germacrene D	0.114	0.109	0.076
Ledene	0.076	−0.121	0.052
α‐Farnesene	0.079	0.230	0.006
β‐Bisabolene	0.170	−0.133	0.167
δ ‐Cadinene	0.154	0.190	0.179
Epiglobulol	0.081	0.185	0.011
(‐)‐Globulol	0.122	−0.008	0.330
Ledol	0.112	0.030	0.315
α‐Bisabolol	0.215	−0.070	0.016
E,E‐Farnesal	0.198	0.072	−0.058
Hexahydrofarnesylacetone	0.148	0.104	−0.035
Hexadecanoic acid, methyl ester	0.155	−0.175	−0.064
Hexadecyl acetate	0.066	0.185	−0.020
Methyl oleate	0.150	−0.011	−0.116
Octadecanoic acid, methyl ester	0.146	−0.177	−0.022
Linoleic acid	0.168	−0.030	−0.080
Methyl octaclecanoate	0.109	0.166	0.151
Oleic acid	0.104	0.239	−0.004
Ethyl Oleate	0.184	−0.118	−0.061
(E)‐9‐Octadecenoic acid ethyl ester	0.192	−0.176	−0.115
Z‐13‐Octadecen‐1‐ol acetate	0.164	−0.209	−0.075
(Z)‐9‐Tricosene	0.136	−0.119	−0.081
Heptacosane	0.084	−0.167	−0.058
Squalene	0.091	−0.100	−0.077
Unknown 1	0.073	0.239	−0.024
Triacontane	0.111	0.031	0.332
Unknown 2	0.123	0.073	−0.134
Eigenvalue	14.895	8.267	6.119
% of Explained variance	24.83	13.77	10.19

Because the availability of blend sources can change temporarily, we compared the blend composition from bees collected in April and June. The differences in number of compounds that were found in the bees collected in April and June was highly significant (*x* ^2^ = 61.05, *df = 32, p *=* *.001). In average, the males collected in April (*n *=* *56) had 20.34 compound in their blends, whereas the males collected in June (*n *=* *44) had an average of 20.2. In April, the males showed more inter individual variation in their blends compounds (CV = 60.15) than in June (CV = 31.35). Moreover, we found highly significant differences in the amount of blends between April and June. The males collected in April have larger amounts of blends (*t *=* *4.69, *df = 98, p *<* *.001). Despite the temporal differences in the blends amount, the PC 1 (*t *=* *1.91, *df = 98, p *=* *.059) and PC 3 (*t *=* *0.21 *df = 98, p *=* *.83) did not differ statistically between collecting dates. Nonetheless, there were significant differences between April and June in the blend composition for the PC 2 (*t *=* *3.95 *df* = *98, p *<* *.001).

### 3.3 Relationship between males’ body size and number of compounds, amount of the blend and blend composition

We did not find any relationship between body size with the number of compound found in the blends in April (*F*_(1,54)_ = 0.11; *p *=* *.74), and June (*F*_(1,42)_ = 0.32; *p *=* *.58), and in the amount of their blends of the males in both, April (*F*_(1,54)_ = 0.54; *p *=* *.46) and June (*F*_(1,42)_ = 0.27; *p *=* *.60). Moreover, neither of the three PC components of the blends was related to body size; PC 1: (*F*_(1,98)_ = 0.48; *p *=* *.48), PC 3: (*F*_(1,98)_ = 3.67; *p *=* *.058), and PC 2: April (*F*_(1,54)_ = 0.11; *p *=* *.74); June (*F*_(1,42)_ = 0.32; *p *=* *.58).

### 3.4 Relationship between PO content and male body size

**Figure 1:**
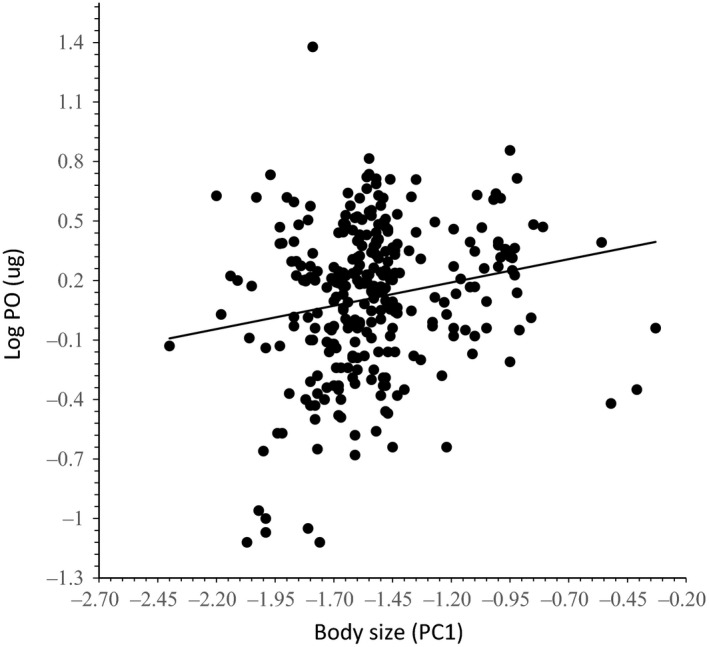
Relationship between phenol oxidase (PO) content and body size (PC 1) in males of *Euglossa imperialis* collected at Los Tuxtlas, Veracruz, Mexico

The regression of PO content on males’ body size (PC1) was positive and significant when we considered all males captured in the field (*r* ^2^ = .06; *F*_(1,273)_ = 18.29; *p *<* *.0001, Figure 1).

### 3.5 Relationship between PO content and blend composition

#### 3.5.1 Relationship between PO and number of compound and the blend's amount

**Figure 2:**
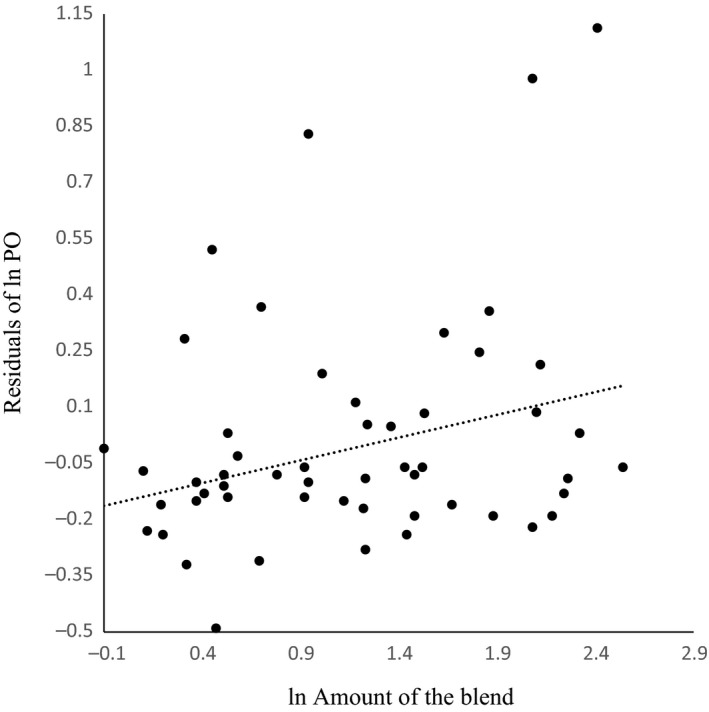
Relationship between the residuals of phenol oxidase (PO) content and the amount of the blend for the males of *Euglossa imperialis* collected in April 2013 at Los Tuxtlas, Veracruz, Mexico

We did not detect a significant relationship between the number of compound in the blend and the residuals PO for the bees collected in April (*F*_(1,54)_ = 0.48; *p *=* *.49), and June (*F*_(1,42)_ = 0.17; *p *=* *.68). In addition, we found a positive relationship between the amount of the blend and the residuals PO for the bees collected in April (*r* ^2^ = .07; *F*_(1,54)_ = 4.47; *p *=* *.04; Figure 2). Nonetheless, in June, this relationship was not significant (*r* ^2^ = .02; *F*_(1,42)_ = 1.01; *p *=* *.32).

#### 3.5.2 Relationship between PO and the PC2 of the blend composition

We did not find a significant relationship between the PC 2 of the blend composition and the residuals PO for the bees collected in April (*F*_(1,54)_ = 0.18; *p *=* *.67), and June (*F*_(1,42)_ = 0.71; *p *=* *.40).

#### 3.5.3 Multiple linear regression analyses

**Figure 3:**
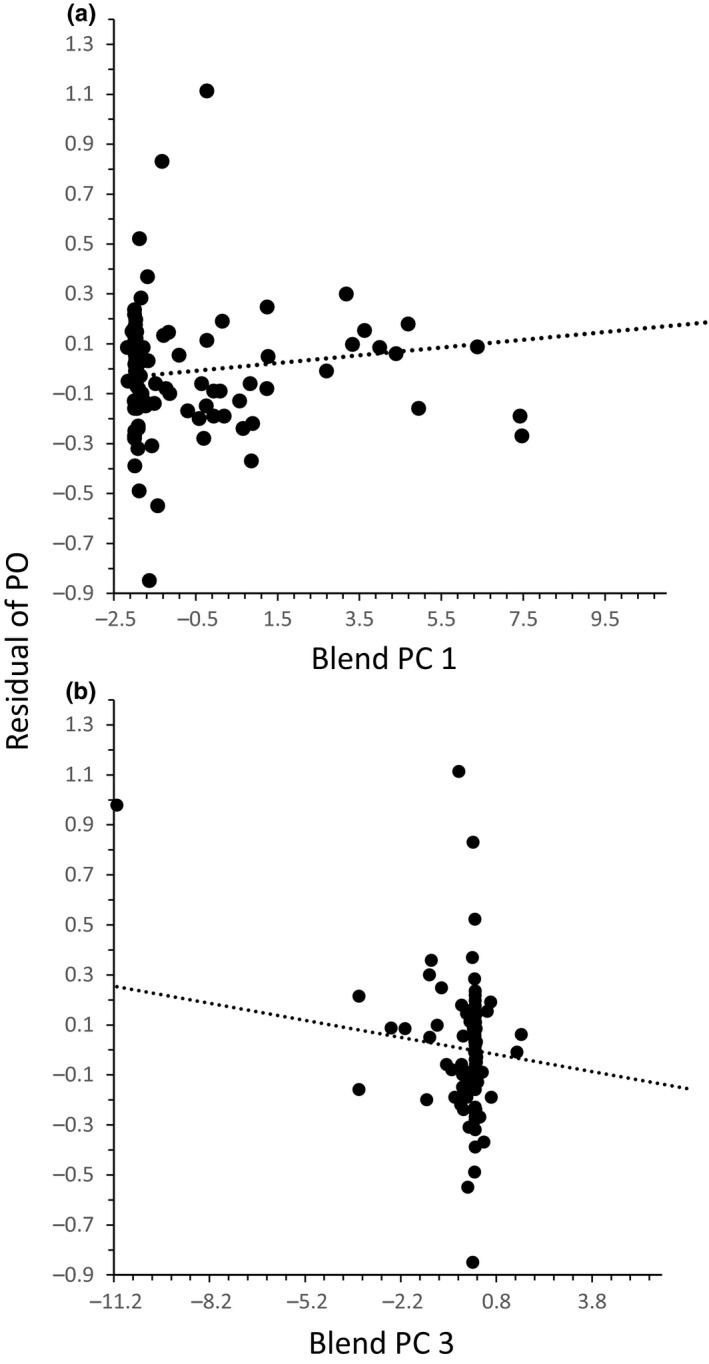
Relationship between the residuals of phenol oxidase (PO) content and (a) PC 1 and PC 3 (b) of the blend composition of males of *Euglossa imperialis* collected at Los Tuxtlas, Veracruz, Mexico

The multiple regression analysis to explore the relationship between the PCs 1 and 3 of blend composition with the residuals of PO content was highly significant (Table 4). The PC 1 and PC 3 of the blends showed positive and negative significant relationships with the residuals of PO content, respectively (Figure 3a,b). Those compounds in the PC1 and PC3 with the highest positive weight were positively correlated with PO levels in the males.

**Table 4:** Multiple regression of PO content on PCs 1 and 3 of the blend composition of *Euglossa imperialis* males collected at Los Tuxtlas, Veracruz, Mexico. *r* ^2^ = .09, *F*_(2,97)_ = 5.03; *p *=* *.008

Source	*df*	S.S.	M.S.	*F*	*p*
PC 1	1	0.349	0.349	5.270	.024
PC 3	1	0.317	0.317	4.788	.031
Error	97	6.422	0.066		

## 4 Discussion

We found that body size, the amount of the blend and its composition (PC 1) are positively related to bees’ immune function in *Euglosasa imperialis*. If females of this species are evaluating their potential mates through their essences, body size and blend composition may represent reliable indicators of male quality. Moreover, we found temporal variation in the number and amount of the compounds in the blends of the bees collected in April and June, which may reflect seasonal differences in compounds availability. Nonetheless, the number of the compounds in the blend and its proportion could not represent the availability of the compounds in the environment. The composition of the blend could be modulated by frequency‐dependent selection; males that collect rare compounds could be preferred by the females. Also the blends may reflect the males’ selective preferences based on experience and learning (Roubik & Knudsen, 2016), and if odors are used to avoid interspecific reproductive interference (see Zimmermann, Ramirez, & Eltz, 2009), the complexity of the blend could be modulated by changes in the community in the number of orchid bee species. Because odor collection and production are costly, the males of *Euglossa imperialis* would be expected to collect a lower number and lesser amounts of compounds in the season with the lower number of orchid bee species (Roubik & Knudsen, 2016).

The composition of the bees’ blends can depend on the availability of the blend sources and the bees’ skills to get access to them. In orchid bees, body size is strongly related to flight capacity (Casey, May, & Morgan, 1985; Darveau, Hochachka, Welch, Roubik, & Suarez, 2005), and large males of *Euglossa imperilis* could displace their competitors for the access to scents (Dworschak & Blüthgen, 2010). Nonetheless, we did not find any relationship between the males’ size with the number, amount and composition of the blends, but we found a positive relationship between the amount of the blends and its composition with PO, which suggest that differences in male condition (immune response) rather than differences in body size may explain the inter‐individual variation in the blends’ composition. The amount of the blend and the proportion of its compounds could reveal the males’ condition to their potential mate, even though, due to environmental fluctuations, perhaps just some compounds can be reliable indicators of the immunological capacity of the males.

Diverse factors can affect the immune response, including aging (Parker, 2010), diet, and energetic reserves (McKean & Nunney, 2001; Singer, Mason, & Smilanich, 2014; Siva‐Jothy & Thompson, 2002; Valtonen, Kleino, Rämet, & Rantala, 2010). In other species, larger individuals can devote more resources to several life history traits including the immune function (Córdoba‐Aguilar et al., 2009; Ryder & Siva‐Jothy, 2001). Nonetheless, the resource allocation to immune function may be subjected to a trade‐off between other life history traits such as reproduction and life span (Schmid‐Hempel, 2011; Sheldon & Verhulst, 1996). In those species that produce pheromones, due to their costs, it is possible to expect trade‐offs with other life history traits. Interestingly, despite the many potential sources of inter‐individual variation in PO content levels, we found large quantities of PO content associated with larger males of *Euglossa imperialis,* suggesting that they are in a better condition than smaller males and they can canalize more energetic reserves to the PO production.

Some compounds collected by orchid bees are involved in the production of pheromones in other insects. In male moths, Farnesal is mixed up with short‐range produced pheromone (Sarto i Monteys et al., 2012), and it might be implicated in the synthesis of marking pheromones of bumblebees (Luxová, Urbanová, Valterová, Terzo, & Borg‐Karlson, 2004), but also it is important in the insect juvenile hormone synthesis (Nyati et al., 2013), in which the insects are involved in females receptivity (Riddiford, 2012) and regulate vitellogenesis (Hansen, Attardo, Rodriguez, & Drake, 2014). On the contrary, Bisabolol (Kamatou & Viljoen, 2010), and β‐Cubebene (Skocibusic & Bezic, 2004) have antimicrobial content. Due to the potential increase in fecundity or their antimicrobial content, perhaps these and other compounds could be valuable nuptial gifts. Moreover, because many of the collected compounds are toxic, the blends also may suggest good genes to the females if males’ resistance to toxic compounds is heritable. The males may show its handicap that could show to females in terms of their endurance to toxicity and their capacity to survive and collect scarce resources (Eltz et al., 1999; Whitten et al., 1989). Our study shows that body size and blend composition are significantly related to PO content in *Euglossa imperialis* orchid bees. This relationship suggests that body size and the blends collected by these bees are honest signals showing the males’ quality to female bees.

## Conflict of Interest

None declared.
