# Corrigendum

**DOI:** 10.1002/ece3.9082

**Published:** 2022-07-11

In the paper published by Arriaga‐Osnaya et al. (2017), the authors reported significant differences in the amount of blends found in *Euglossa imperialis* between April and June. However, this result is incorrect. After reanalyzing the differences between April and June in the amount of blends, the result is not significant (*t* = 0.99, g.l. = 98; *p* = .32).

The authors apologize for this oversight.
